# The impact of short videos on student performance in an online-flipped college engineering course

**DOI:** 10.1057/s41599-022-01355-6

**Published:** 2022-09-22

**Authors:** Jia Zhu, Hang Yuan, Quan Zhang, Po-Hsun Huang, Yongjie Wang, Sixuan Duan, Ming Lei, Eng Gee Lim, Pengfei Song

**Affiliations:** 1 Department of Mechatronic Engineering, Suzhou City University, 215104 Suzhou, China; 2 School of Advanced Technology, Xi’an Jiaotong-Liverpool University, 215000 Suzhou, China; 3 Department of Electrical and Electronic Engineering, University of Liverpool, Liverpool, UK; 4 Department of Mechanical Engineering, Massachusetts Institute of Technology, Cambridge, MA USA; 5 School of Science, Harbin Institute of Technology-Shenzhen, 518000 Shenzhen, China

**Keywords:** Education, Science, technology and society

## Abstract

The 2020 COVID-19 pandemic has greatly accelerated the adoption of online learning and teaching in many colleges and universities. Video, as a key integral part of online education, largely influences student learning experiences. Though many guidelines on designing educational videos have been reported, the quantitative data showing the impacts of video length on students’ academic performance in a credit-bearing course is limited, particularly for an online-flipped college engineering course. The forced pandemic lockdown enables a suitable environment to address this research gap. In this paper, we present the first step to examine the impact of short videos on students’ academic performance in such circumstances. Our results indicate that short videos can greatly improve student engagement by 24.7% in terms of video viewing time, and the final exam score by 9.0%, both compared to the long-video group. The quantitative Likert questionnaire also indicates students’ preference for short videos over long videos. We believe this study has important implications for course design for future online-flipped engineering courses.

## Introduction

Remote online learning and teaching (RO-L&T) has long been considered as a promising education practice in the future, thanks to its wide availability and timing convenience. The 2020 COVID-19 pandemic has greatly accelerated the usage of RO-L&T in many universities (Adedoyin and Soykan, 2020; Adnan and Anwar, 2020; Ali, 2020; Aristovnik et al., 2020; Besser et al., 2020; Fatonia et al., 2020; Fresen, 2018; Li et al., 2022; Radha et al., 2020), due to the forced campus shutdown. This brings new challenges to both students and instructors, though some RO-L&T experiences have been accumulated in the last two decades (Bach et al., 2006; Keengwe and Kidd, 2010). To foster a RO-L&T environment, there are mainly three existing methods (Bergmann and Sams, 2012; Hrastinski, 2008; Yamagata-Lynch, 2014; Young et al., 2014): (1) synchronous RO-L&T, where the instructor and students gather at the same time, and a live video lecture is delivered; (2) asynchronous RO-L&T, where the instructor uploads the pre-recorded videos, and the students study those materials at their own time; and (3) flipped RO-L&T.

Among them, the flipped RO-L&T has unique advantages, thanks to its promotion of student-centered activities, including discussions, presentations, interactive feedback, and critical thinking exercises (Bognar et al., 2019; Caviglia‐Harris, 2016). It is even more advantageous when combined with educational videos. In a flipped RO-L&T, the pre-recorded videos are learned at students’ own pace before the synchronous flipped class session, where the student-centered dynamic and interactive L&T activities are conducted (Galway et al., 2014; Jia et al., 2020). Under this format, students are usually more motivated to learn, thereby generating more teacher-student interaction than that in the traditional classes, which increases the efficiency of teaching and learning (Cristina Blasco et al., 2016; Moos and Bonde, 2016). The videos are used as the primary study materials for student self-studying. As a result, the pre-recoded videos play a significant role in determining the effectiveness of an online-flipped course and thereby the student learning outcome (Guo et al., 2014; Meseguer-Martinez et al., 2017; Slemmons et al., 2018). Video can create an immersive learning atmosphere that help students remember key knowledge and develop critical thinking (Mora, 2016). Moreover, the promotion of online platforms has further popularized the flipped RO-L&T, enabling more affordable and user-friendly classroom instruction (Slemmons et al., 2018). Its use in language education has been shown that is able to improve students’ language proficiency, facilitate practice, and enhance learning outcomes (Gloudeman et al., 2018; Lopes and Soares, 2016; Özkurkudis and Bümen, 2019). However, those results need to be further quantified in more disciplines.

In most flipped classrooms, videos are effective for teaching and learning (Robinson et al., 2020). Many video factors are related to the overall course effectiveness in RO-L&T (Christ et al., 2017; Guo et al., 2014). These include but are not limited to video length, recording location, instructors’ speaking speed, and image (Guo et al., 2014). Among all the factors, video length is one of the most straightforward influencing factors, which can be quantitatively studied (Brame, 2016; Slemmons et al., 2018). Theoretically, short videos are preferred because they are flexible, convenient, engaging, and popular with students (Long et al., 2016; Yang, 2017). As explained in cognitive load theory and cognitive theory of multimedia learning, the short video can reduce extraneous cognitive load and thus aid in schema construction (Chandler and Sweller, 1991; DiMaggio, 1997). Guo et al. (2014) harvested student data from massive open online courses (MOOC) and statistically concluded that short (i.e., 6– 10 min) videos have longer student viewing time than that of long videos (>12 min). Slemmons et al. (2018) organized a flipped K-12 class in a middle school and found that short videos are more engaging for students but no quantitative improvements on exam scores were founds.

The benefits of short videos in flipped classes have also been demonstrated in many other prior studies (Brooks, 2014; Diwanji et al., 2014; Slemmons et al., 2018; Zuber, 2016). Most documented studies are organized either (1) in a non-credit-bearing course (i.e., MOOC) (Li et al., 2015; Wang and Zhu, 2019), or (2) in a campus-based environment where an in-person class is used for interactive activities (Rui et al., 2017; Zuber, 2016). The flipped class that is completely held in RO-L&T has rarely been studied in a college credit-bearing course. That could be partially due to the seriousness of final degree-awarding. Most recently, because of the forced campus lockdown by the COVID-19 pandemic, a fast-growing number of studies on RO-L&T flipped college courses have been reported (Jia et al., 2020; Marshall and Kostka, 2020; Nerantzi, 2020; Tang et al., 2020; Yen, 2020). The lockdown enables a good research environment to quantitatively and qualitatively study RO-L&T, which may shed the light into the future education.

In this study, we present a quantitative and qualitative report to examine the impact of short videos on student performance in an online-flipped college engineering course under COVID-19. Engineering drawing is one fundamental course for engineering students. Our primary research question is whether short videos can benefit the academic performance of students in an online-flipped college engineering course. The quantitative and qualitative data are extracted from formal exams and Likert scale questionnaires, and this paper is organized as follows. The literature background, which includes previous related studies and theoretical background, will be described in Sect. “Literature background”. The research hypothesis will be described in Sect. “Research hypothesis”, followed by the case design introduction in Sect. “Engineering drawing case study”. The quantitative and qualitative data, with the statistical analysis discussion, are then presented in Sects. “Results” and “Discussion”. Finally, the recommendation and conclusion are given in Sects. “Recommendations” and “Conclusion”.

## Literature background

### Flipped and conventional asynchronous online learning and teaching

In the last decade, the flipped classroom has become a new learning and teaching format that is widely studied and adopted by many higher education institutions worldwide (Bergmann and Sams, 2012). Since the outbreak of the COVID-19 pandemic, the adoption of flipped RO-L&T has become increasingly important (Rad et al., 2021; Yen, 2020). Teachers provide pre-recorded instructional videos for students to watch whenever they are free (Chick et al., 2020). Internet communication tools and platforms in this teaching model became the medium to ensure teacher-student interaction, similar to asynchronous RO-L&T (Chick et al., 2020).

However, the most important difference between flipped RO-L&T and asynchronous RO-L&T is the shift of student identity from passive to active in flipped RO-L&T. In asynchronous RO-L&T, the students act as auditors, passively receiving information from the video but often ignoring the process of learning activities such as discussions and peer reviewings (Guo et al., 2014; Kizilcec et al., 2013; Phillips and Wiesbauer, 2022). In contrast, in flipped RO-L&T, students are the dominant roles and actively engage in student activities like group discussions. These make the class more engaging for meaningful discussions and idea exchanges. This shift has consequent implications in terms of knowledge retention, learning outcomes, and students’ motivation and engagement (Afzali and lzadpanah, 2021; Shatto et al., 2017; Yu and Gao, 2022).

### Effect of video lengths

After the pandemic begins, the increasing use of RO-L&T challenges students’ attention to videos (Revadekar et al., 2020). Long videos of the same length as that in the traditional in-person class (about 50 mins) are usually not attractive to students, directly affecting students’ academic performance (Li et al., 2021; Turan-Özpolat, 2020). Therefore, more studies are beginning to discuss the impact of video length on learning and teaching, to optimize the flipped classroom effectiveness and student experience (Afify, 2020; Manasrah et al., 2021; Slemmons et al., 2018; Yu and Gao, 2022).

Afify (2020) tested the effect of three different video lengths (short <6 min, medium 6–12min, and long >12 min) on student performance, and found short lecture videos can lead to better academic achievement. Manasrah et al. (2021) obtained similar results and concluded short videos are more entertaining and informative. In addition, another study has shown that students are less engaged with long videos, often viewing them for a short period of 5–6 min, reflecting a tendency for shorter videos (Kuznekoff, 2020). The online-flipped classroom, with the assistance of length-optimized videos (short videos), can stimulate students’ motivation and independent learning, ultimately improving teaching effectiveness (Chang et al., 2015; Yang, 2017; Yin and Liu, 2017). Therefore, it is necessary and meaningful to further explore the impacts of video length quantitatively in flipped RO-L&T.

### Short-term and long-term knowledge retention

Knowledge retention focuses on transferring new information from short-term retention to long-term retention. The instructional model will significantly impact students’ knowledge retention, affecting academic performance (Mithun and Evans, 2018). In traditional classrooms, especially in some credit courses, teachers are required to lecture hundreds of students and deliver large amounts of knowledgeable information within 1–2 h (Gannaway et al., 2018; Huxley et al., 2018). Students are often too busy taking notes to think and digest, resulting in only short-term and low knowledge retention (Hadie et al., 2019).

Recently, several studies have demonstrated that the flipped RO-L&T can increase knowledge retention by motivating students through enriched student interaction activities such as questioning, discussions, and idea exchanges (Estes et al., 2014; Hawks, 2014; Kerr, 2015; Shatto et al., 2017). More specifically, short-term retention (~3 months) is much higher in traditional teaching method, while long-term retention (~12 months) is obviously enhanced under the flipped teaching approach (Shatto et al., 2017). In a case study performed in the introductory programming course (Mithun and Evans, 2018), the flipped classroom improved the students’ knowledge retention and average grades in the end.

### Students’ engagement and motivation

Student engagement reflects the subjective willingness of students to participate in activities such as lectures and in-class exercises. Low engagement negatively impacts learning and academic performance (Wang et al., 2014). In contrast, high engagement (active learning) usually enhances learning experiences and outcomes (Ferrer et al., 2020). Motivation is usually an individual’s drive to accomplish something (i.e., the degree of desire to continue pursuing it) (Wen and Piao, 2020). It is explicitly related to engagement, which leads to or facilitates engagement and is a core principle of teaching and learning (Dabbagh, 2007; Lee and Reeve, 2012; Mitchell, 2011; Reeve, 2012; Skinner et al., 2008).

In terms of engagement and motivation, different teaching modes can bring significant different effects, which are even more influential for RO-L&T compared to on-campus students (Bawaneh and Moumene, 2020; Ferrer et al., 2020; Kamarzaman et al., 2022). Afzali and lzadpanah (2021) selected 360 English language learners, and they were randomly assigned to the control and experimental groups. Data were collected using a questionnaire during a six-week English course, and a significant increase in students’ engagement and motivation was found for the flipped classroom.

## Research hypothesis

Based on the research question, we developed two research hypotheses. The first hypothesis tests the video engagement time (defined as the average student video viewing time per video) difference between the short-video group and long-video group. The second hypothesis tests the quantitative exam scores between the two groups.

### Hypothesis 1

H_0_ For the online-flipped engineering drawing course, the student video engagement time of the short-video group and long-video group are identical.

H_1_ For the online-flipped engineering drawing course, the student video engagement time of the short-video group is longer than that of the long-video group.

### Hypothesis 2

H_0_ For the online-flipped engineering drawing course, student exam scores of the short-video group and long-video group are identical.

H_1_ For the online-flipped engineering drawing course, student exam scores of the short-video group are better than that of the long-video group.

## Engineering drawing case study

### Design of the online-flipped course

**Fig. 1:**
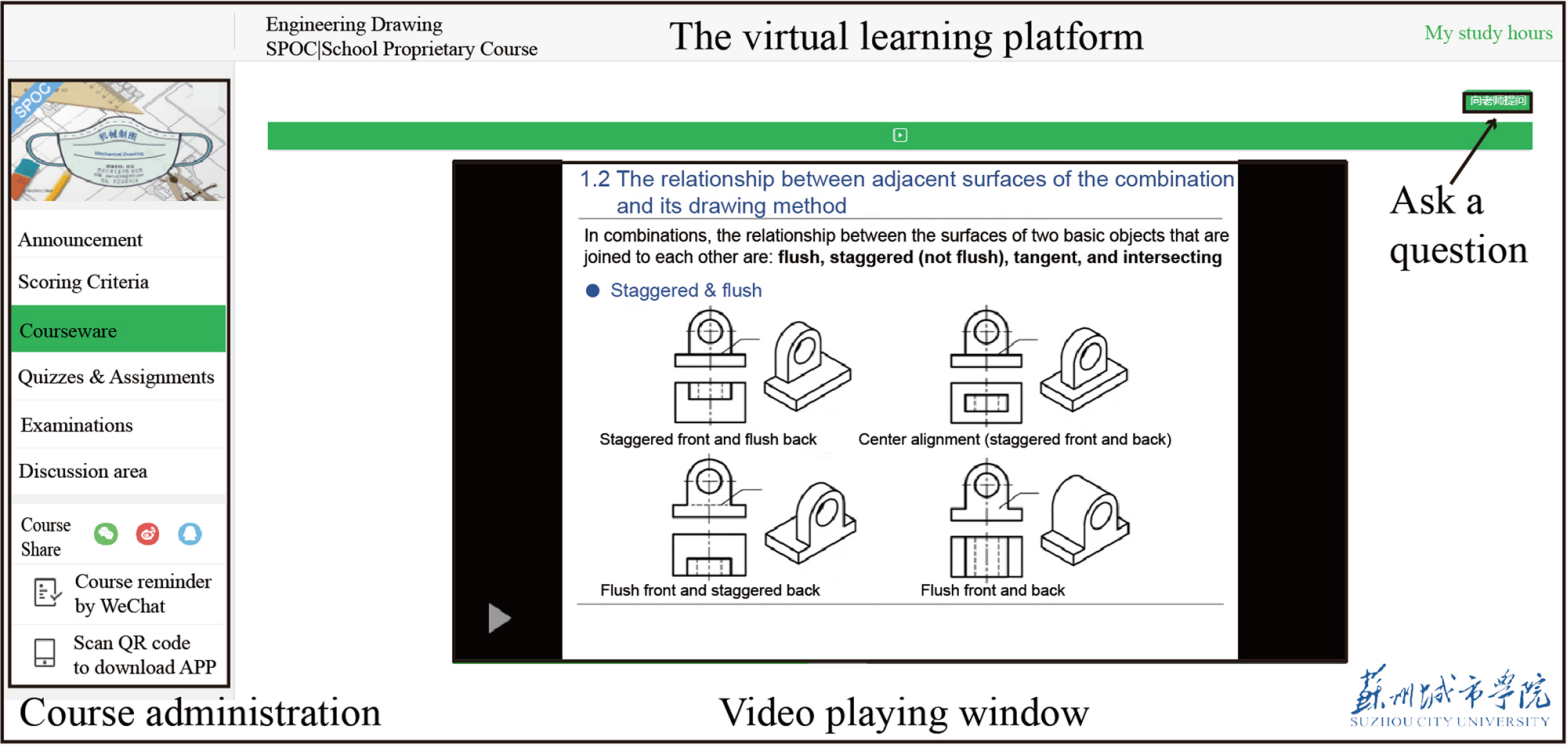
The virtual learning and teaching platform. Screenshot of the platform and video viewing activities.

This case study was conducted in the *Introduction to Engineering Drawing* course at Suzhou City University. The practice of flipped classes was designed according to Bishop and Verleger (2013), but all online. Briefly, the pre-recorded lecture videos, along with other materials, were uploaded to the virtual learning platform (Fig. 1). Students usually have one week to learn those materials before the 2-hour interactive classroom. The video viewing behavior for each student, including the number of clicks and total viewing time, is automatically recorded. During the 2-hour interactive classroom, student-centered activities such as group discussions, tutoring, and questions & answers are conducted. The platform is also used to collect homework after class.

### Student population

This course is mandatory for all freshmen students majoring in mechanical engineering at our university. They all have a similar level of academic performance in China’s national college entrance examination (or Gaokao). To rule out the influences of prior knowledge, the repeating students are not included in this study. Descriptive statistics of selected background information of the participating student group are shown in Table 1. Briefly, there are 65 students in total, and 11 of them are female. There are significantly more male students than female students in engineering majors, such as mechanical engineering (this study), electrical and electronics, computers, and automatization. This is why the male participants in this study greatly outnumbered the females.

**Table 1:** Descriptive statistics of the participating student group.

	Long video group	Short video group	Total
No. of student participants	35	30	65
No. of females	6	5	11
No. of males	29	25	54
Minority students	0	0	0
Disabled students	0	0	0
Average of Gaokao scores^a^	317.9 ± 7.6	319.3 ± 7.8	317.9 ± 6.8

^a^The total mark of Jiangsu Gaokao is 480.

The intrinsic learning skill of students that are developed from early studies may also influence the results of this research, but that skill can be well-evaluated based on the Gaokao score (Bai and Chi, 2011; Bai et al., 2014). Our participants were all admitted from Jiangsu Province and have taken the same Gaokao exam sheets, allowing us to safely rule out the score differences contributed by different provinces. Our college usually only admits Tier-2 students with similar Gaokao scores, and significant score differences are not observed (Table 1). The standard deviation of students’ Gaokao scores is less than 2.2%, indicating no significant difference in intrinsic learning skills exists among the participating students.

In addition, in our case study, the students had not been informed about pre-knowledge, such as “student preferences for short videos compared to long videos,” when they started this case study. Our questionnaire asking about the “preferences” was conducted at the end of the case study, so the pre-bias from the students should not play roles in the study. Moreover, the short and long-video student groups were randomly formed to eliminate the potential biases of different individuals. Though we cannot be completely sure that the bias was eliminated, similar approaches have been used in many previously published studies (Afify, 2020; Manasrah et al., 2021; Slemmons et al., 2018; Yu and Gao, 2022).

### Experimental design

Our registry department randomly assigned the student groups: 35 students participating in the long-video group and 30 students participating in the short-video group. The average Gaokao scores for the long and short-video groups are 317.9 ± 7.6, and 319.3 ± 7.8, respectively. Such a small difference (less than 0.1%) indicates that both groups share similar learning skills.

We pre-recorded weekly lecture videos via the video recording function that comes with the online virtual platform, with a normal length (~55 min, same as the length of the regular in-person class in our college), and they were directly used for the long-video group. The teaching style is that a teacher shows slides while lecturing. The short video was created by trimming the long-video into the videos with average 8-mins long (Editing platform: Adobe Premiere Pro, video resolution: 720 p, video coding format: H.264, audio format: mp4), which has been proved to the optimal video length (Guo et al., 2014).

As engineering courses typically revolve around the establishment of a conceptual system and the acquisition of engineering skills, the knowledge points are many but systematic, and can be identified based on the concept or skill point. For example, when lecturing engineering drawing courses, the knowledge points such as perspective view, three views, and oblique diagraming of the engineering drawings can be easily and clearly segmented into short videos (Jian, 2011; Zuo et al., 2003). In short, the long videos are produced based on the content of the textbook chapters. They are long enough to contain one complete chapter. For short videos, the completeness and consistency of the teaching content (one knowledge point) are still considered, though it was cut from the long videos, and the length usually ranges from 7 min to 9 min (average 8 mins). Though short, these videos present at least one complete knowledge point. Limited by the current technology, the system was unable to tell or monitor whether or not all students in each group were sitting in front of their computers and watching the video when the video was playing (Deepa et al., 2022; Gupta, 2022), but the students were explicitly told that this kind of behavior was prohibited.

The pre-course and post-course questionnaire surveys are used to collect students’ perceptions before and after the course, respectively. One final exam is used to test the two groups quantitatively, and it is scheduled at the same time with a two-hour duration with online camera invigilation.

### Questionnaire design

The five-point Likert questionnaire contains 9 questions, each followed by a scale: *A. strongly agree, B. agree, C. neutral, D. disagree*, and *E. strongly disagree*. The first 3 questions are used to quantitatively understand students’ perceptions and attitudes on the RO-L&T before the class begins. By understanding that, we can analyze the influences brought by the students’ intrinsic attitudes. The remaining 6 questions were answered after the class ended. They were used to know how the students watched the videos and their perspectives on the short and long videos. The Cronbach’s alpha test was used to assess the reliability of the survey and indicated a high degree of reliability and internal consistency of our survey (*α* = 0.87).

### Statistical analysis

All statistical analyses were performed using Matlab (MathWorks Inc). Cronbach’s alpha test was used to examine the reliability of the Likert scale survey. Both groups’ exam scores and video viewing time were compared using the Mann–Whitney *U*-test, due to the rejection of the normality test. The significance level was set to 0.05 for checking the hypotheses. The Likert survey data were compared using a one-way ANOVA test.

## Results

### Short video is more engaging

**Fig. 2:**
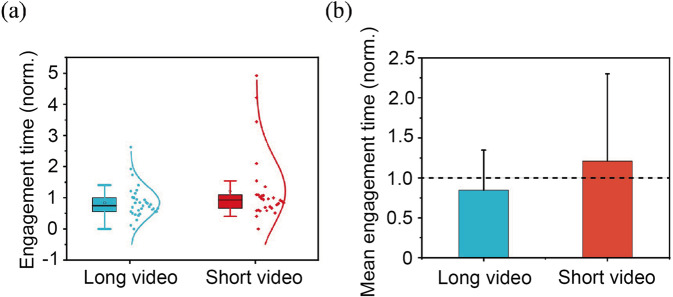
The engament time for both groups. **a** Average engagement time of each student. In the box, the middle black bar is the median; the top and bottom bars are 25th and 75th percentiles, respectively. The normal distribution fitting of the data set is also shown along the right side of the boxes. **b** The normalized mean engagement times for long-video and short-video groups.

By far, video viewing time (engagement time) has been considered as the most significant indicator of student engagement in online education (Guo et al., 2014). Statistically, data normalization is often used to compare data from different groups. Therefore, we scaled each student’s average engagement time of the long and short-video groups to fall into the range of 0 to 1. Especially, students who repeatedly watch the video several times will result in a normalized value greater than 1, which indicates the total viewing time has exceeded the total video length. The boxplots (Fig. 2a) show the short-video group has much fewer variances of engagement time, and more than 70% of students watched over three-quarters of the video length. A 24.7% improvement in median engagement time was also demonstrated with the normalized data (0.932 for the short-video group and 0.747 for the long-video group). These results suggest that short-video length significantly increases student engagement, and our hypothesis 1 can be validated.

The improvement can also be seen in terms of the normalized (Fig. 2b) engagement time for the two groups. The normalized value for the long-video group is smaller than 1. In contrast, the value for short is larger than 1, suggesting more student engagement. Our findings agree with the prior study performed on the MOOC platform and suggest that short videos are also more engaging for students under remote online teaching (Guo et al., 2014). Additionally, both groups have led to enhanced engagement time compared to that of MOOC, which may be attributed to the credit-bearing nature of this course.

### Short video enhances the academic performance of students

**Fig. 3:**
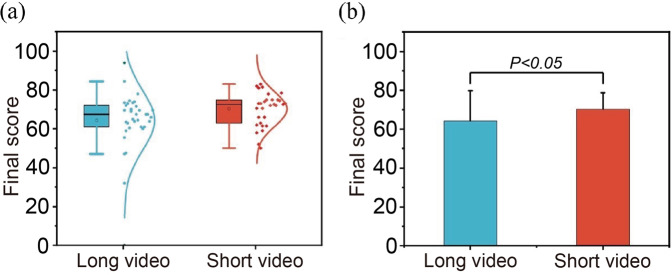
The exam scores for both groups. **a** Boxplots of the final exam scores for both groups, and **b** average final exam scores for both groups.

The Mann–Whitney *U*-test was used to evaluate the dependence of exam scores on video length (López et al., 2015). Figure 3a shows the boxplots of final exam scores for both groups. In detail, the short-video group outperformed the long-video group in many quantitative metrics. The median and average exam scores of the short-video group are, respectively, 7.4% and 9.0% higher than those of the long-video group. The short-video group also shows a smaller standard deviation, indicating that the students may have performed more consistently. Figure 3b shows that the *p*-value of 0.0334 (<0.05) for the average score comparison was obtained. This result demonstrates that short videos can result in better academic performances, indicating the validation of our hypothesis 2.

### Results description

Based on the above quantitative results, several findings and contributions that are different from previous studies can be discussed and summarized below.

First, compared to those studies on non-credit MOOC courses (Guo et al., 2014; Moore and Blackmon, 2022), our study is built on credit-bearing engineering courses. Students have different attitudes and behaviors toward the two types of courses, while the credit-bearing courses usually receive much more attention (Joyner, 2022; Lohse et al., 2020). It is also different from language flipped classes (e.g., English) (Al-Assaf et al., 2022; Demyanova, 2022; Yu and Gao, 2022), which focus on training language skills such as grammar and oral expression. Engineering courses focus on knowledge point acquisition and engineering skill improvement, which are more easily divided into several individual short videos. Different subjects have different effects on students’ learning behaviors and habits (Kerr, 2015; Young et al., 2014; Yu and Gao, 2022). Such differences may generate new findings. Therefore, it is still meaningful to perform one case study on video usage in this environment and make new contributions to community research on teaching and learning.

Second, compared to studies where students only watch videos and skip activity sessions such as assessment and discussion (Guo et al., 2014; Kizilcec et al., 2013), in our study, students are the dominant actors in student-centered activities, including discussion, presentations, and interactive feedback. Students’ identities in learning activities affect students’ motivation and engagement.

Third, compared to studies in which engagement was measured through students’ subjective responses to engagement questions (e.g., cognitive, behavioral, emotional, and social) (Yu and Gao, 2022), we measured engagement objectively by the time spent watching videos (engagement time). Moreover, as shown in Sect. “Short video enhances the academic performance of students”, we added one quantitative assessment metric, which assessed the increase or decrease in student learning performance based on final exam scores.

## Discussion

### Learning influencing factors

RO-L&T has long been considered as a promising education practice with the great potential to eliminate education inequality. The pandemic lockdown provides the perfect opportunity to construct an RO-L&T environment to perform this pedagogical research. Our results demonstrate that with short videos, students tend to be more engaged with better exam scores in the flipped RO-L&T environment. We have to admit that learning is a complicated process, and many influencing factors are involved, such as students’ attitudes, learning skills, and prior experiences. Using the one-way ANOVA test, the influences of these factors were also examined based on the Likert scale questionnaire.

### Student internet skills and subjective attitude

**Fig. 4:**
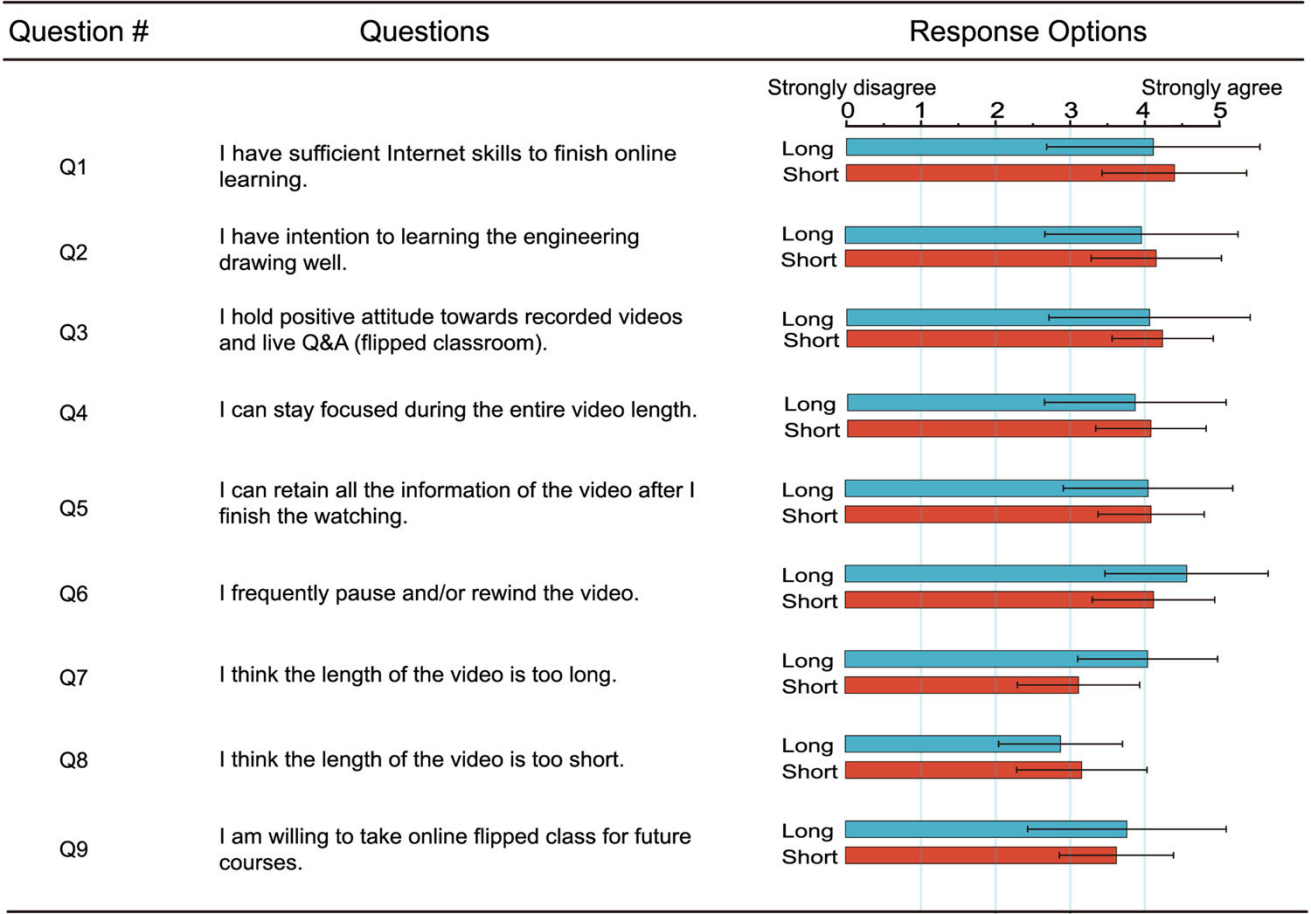
Average scores of both groups for each questionnaire survey question.

Although RO-L&T has been developed for a while, students may still be limited by their internet skills to adapt to the flipped RO-L&T. As students’ negative subjective attitudes toward the learning content (engineering drawing) and learning format (recorded video and flipped classroom) may also affect the experimental results. Therefore, we conducted a data survey on students’ internet skills and subjective attitudes through three questions (Q1-Q3) before the class to exclude the influence of subjective factors and large individual differences. As shown in Fig. 4, there are no significant differences between the two groups in terms of internet skills (Q1, *p* > 0.05), and subjective attitudes (*p* > 0.05 for Q2, *p* > 0.05 for Q3).

### Concentration and information retention

Students’ concentration and self-perceived information retention for videos of different lengths may influence the objective feedback on the experimental results (final exam grades). One interesting finding is that both groups reported a similar level of ability to (1) stay focused for the entire video length (Q4, average score: 3.86 vs. 4.06, *p* > 0.05) and (2) retain the video information (Q5, average score: 4.06 vs. 4.10, *p* > 0.05). However, the objective scores of the final exams showed that significant information retaining differences existed between the two groups. This may suggest that our students all have a good learning attitude (in line with Q2) and wish to stay focused for the entire video length and retain all the information.

### Pause or rewind and length perception

Although we divided the videos into short (8 min) and long (55 min), short and long are relative concepts that also depend on the perception of different students. One unique advantage of using recorded videos is that the students can frequently rewind or pause the video, which can help student process and understand the difficult content and aid the test. This will help eliminate potential result bias caused by difficulties in understanding long videos. The long-video group also reported a higher frequency of video rewinding than that of the short-video group (Q6, average score: 4.58 vs. 4.13, *p* > 0.05), though no statistical difference was identified. This result was also in line with subjective reports when asked if the videos were too long or not, and the long-video group reported a much higher value (Q7, average score: 4.06 vs. 3.13, *p* > 0.05). However, for the question asking if the videos are too short, the short-video group reported a similar value compared to the long-video group (Q8, average score: 3.16 vs. 2.89, *p* > 0.05*)*.

### Findings and limitations

While there were limitations of the current study (e.g., the current technology is unable to verify if students actually watched videos), this study is the first step to assessing the impact of short videos on students’ performance in an online-flipped college engineering course during the pandemic. The strict lockdown enabled us to conduct this credit-bearing course in the RO-L&T environment, which is impossible otherwise. Several new findings can be summarized. First, the engagement time for the long-video group has significantly increased compared to previous MOOC studies. The median engagement time is less than 4 min for 12–40 min long videos in MOOC but increased to 24.7 min for 55-min-long videos in our studies. This suggests that students may be strongly motivated to control themselves for studying towards their degrees and thereby leaves more room for instructors to edit the video length to meet the specific needs better instead of strictly limiting the video length to 6 min that as suggested by MOOC studies (Guo et al., 2014; Yu and Gao, 2022). Second, we experimentally demonstrated that short videos could positively impact learning outcomes in the RO-L&T environment regarding the final exam scores. The cognitive load theory can explain this well: short videos are helpful for students to categorize and compress information into organized compartments (Chase and Simon, 1973; Slemmons et al., 2018). It is also particularly useful to reduce the extraneous cognitive load, enhancing the student’s ability to focus on the germane cognitive load.

### Future work

Next, we will investigate more detailed behavioral parameters that can reflect video effectiveness, information retention, and learning outcomes. For example, students’ opinion of video quality, accessibility (e.g., the potential need to charge devices while watching long videos), preferred viewing device used (computer or phone), viewing location (home, office or commuting), viewing time (day or night), audio playback method (headphones or speakers), preferred video speed (slow, regular or fast) and multitasking (e.g., taking notes while watching). In the following study, we plan to add more qualitative research, such as designing a scale to measure learning activities for improving the videos. In addition to the length of the videos, we will consider quizzes, assignments, multimedia, and other influencing factors when designing videos in the future. As the video platform develops, we may focus on monitoring pre-screen student behavior, absences, and poor interactions, as well as quantitative research on other possible group factors (e.g., gender and personality). Moreover, to enhance external validity and further longitudinal depth, we intend to replicate our experiment in additional engineering courses for follow-up studies.

## Recommendations

“Tell students and they forget, teach students and they may remember, involve students and they learn,” is the core of the flipped classroom concept. Rapid advances in technology have made better implementation of flipped RO-L&T possible. Such an environment is a positive experience for both the instructor and the students while increasing students’ engagement. The evolving engineering courses could benefit from providing length-optimized videos to students under the flipped course structure. In this way, class time can be focused on discussing problems encountered in the videos and on learning how to “think like an engineer”. Flipped RO-L&T may initially be accepted by teachers and students, and the opportunity to improve the instructional model should not be overlooked.

In particular, educators need to use research-proven methods that can be replicated from one course, such as engineering drawing, to another. We offer several recommendations based on this study that we hope these will help instructors improve flipped RO-L&T. First, it is recommended that teachers should make the videos as short as possible, but one complete knowledge point should be contained in one video. Second, all pre-class video preparations are recommended to be linked to student in-class activities, which will help increase students’ motivation and engagement. For example, ask targeted questions related to the video content to provoke active thinking or discussion among students. Third, during the class, according to cognitive load theory, it is recommended to intentionally pause after reviewing each point in the video, to give students time to digest the contents. For example, give three to five minutes of break to allow students to share the things they are unclear about. Fourth, based on our research finding that students are likely to watch the videos repeatedly after class, it is recommended that use one short video to cover one question or knowledge point, rather than one large block of video covering all the questions and points.

## Conclusion

We have presented one study to evaluate the impact of short videos on students’ performance in an online-flipped college engineering course during the COVID-19 pandemic. This credit-bearing course is completely delivered in a remote online learning and teaching environment. Our findings demonstrate that short videos can significantly improve students’ engagement by 24.7 % (median value) and their final exam scores by 7.4%, compared to the long-video group, which can be attributed to the reduced cognitive load. We also discovered that the student engagement time is longer than in MOOC studies, leaving more room for instructors to construct videos in credited course teaching. We are hopeful for future developments and have made some recommendations, including video design, students’ motivation and engagement, knowledge retention, and classroom activities. We envision that our studies could provide useful information for future curriculum design in remote online-flipped courses.

## Data Availability

The data supporting the findings of this study are available upon request from the corresponding author.
